# Distinct brain regions are affected by neurodevelopmental or pre-dementia changes in Down syndrome

**DOI:** 10.1093/braincomms/fcag269

**Published:** 2026-07-13

**Authors:** Lília Jorge, Joana Oliveira, Ricardo Martins, Tânia Lopes, Hugo Quental, Miguel Castelo-Branco

**Affiliations:** Institute for Nuclear Sciences Applied to Health, University of Coimbra, 3000-548 Coimbra, Portugal; Faculty of Science and Technology, University of Coimbra, 3030-790 Coimbra, Portugal; Coimbra Institute for Biomedical Imaging and Translational Research, 3000-548 University of Coimbra, Coimbra, Portugal; Institute for Nuclear Sciences Applied to Health, University of Coimbra, 3000-548 Coimbra, Portugal; Coimbra Institute for Biomedical Imaging and Translational Research, 3000-548 University of Coimbra, Coimbra, Portugal; Institute for Physiology, Faculty of Medicine, University of Coimbra, 3000-548 Coimbra, Portugal; Institute for Nuclear Sciences Applied to Health, University of Coimbra, 3000-548 Coimbra, Portugal; Coimbra Institute for Biomedical Imaging and Translational Research, 3000-548 University of Coimbra, Coimbra, Portugal; Institute for Nuclear Sciences Applied to Health, University of Coimbra, 3000-548 Coimbra, Portugal; Coimbra Institute for Biomedical Imaging and Translational Research, 3000-548 University of Coimbra, Coimbra, Portugal; Institute for Nuclear Sciences Applied to Health, University of Coimbra, 3000-548 Coimbra, Portugal; Coimbra Institute for Biomedical Imaging and Translational Research, 3000-548 University of Coimbra, Coimbra, Portugal; Institute for Nuclear Sciences Applied to Health, University of Coimbra, 3000-548 Coimbra, Portugal; Coimbra Institute for Biomedical Imaging and Translational Research, 3000-548 University of Coimbra, Coimbra, Portugal; Institute for Physiology, Faculty of Medicine, University of Coimbra, 3000-548 Coimbra, Portugal

**Keywords:** Down syndrome, structural MRI, neurodevelopment, episodic memory, Alzheimer’s disease

## Abstract

Down syndrome, a condition characterized by triplication of chromosome 21, leads to a complex interplay between neurodevelopmental and dementia-related changes similar to the ones observed in Alzheimer’s disease. Here we aimed to understand this interplay by using imaging biomarkers for different cognitive profiles in Down syndrome, and by analysing early developmental differences versus age-related changes. We analysed voxel-based morphometric measures of grey matter volume from high-resolution T1-weighted MRI in 23 adults with Down syndrome (18–59 years, five female) in preclinical/prodromal stages of Alzheimer’s disease and 24 age- and sex-matched controls, along with cognitive assessments. Neuroanatomical group differences were assessed using two-sample t-tests. Age-related effects on brain integrity, and cognitive function were examined through voxel-wise regression analyses and correlation tests, respectively. Finally, structural correlates of episodic memory were explored across the whole brain at the voxel level within the Down syndrome group. Results revealed a neuroanatomic phenotype with both regional increases and decreases in grey matter volume compared to controls (false discovery rate, *q* ≤ 0.05). Based on regression analysis, we found the following patterns in regions that were differentially reduced in Down syndrome: same intercept and different age-related slope (defining specific age-related differences), different intercept (implying initial neurodevelopmental differences) and same slope (signalling no age-related differences). A notable example of the first was the left hippocampus and its subfields, and of the second was the orbitofrontal cortex. Follow-up whole brain analyses confirmed age-related changes in Down syndrome (false discovery rate, *q* ≤ 0.05) in the parietal and temporal cortices, extending into hippocampus, as compared to controls, independent of neurodevelopmental (non-age related) features, and most pronounced in the right hemisphere. Episodic and associative memory declined significantly with age (*P* = 0.016) in Down syndrome and correlated with shrinkage in regions vulnerable to Alzheimer’s disease (*P* < 0.01), including the precuneus and posterior cingulate cortex. Our findings suggest that individuals with Down syndrome undergo early brain atrophy that occurs independently of baseline neurodevelopmental changes, particularly in the hippocampus and temporoparietal regions. Notably the posterior cingulate cortex and precuneus showed an association with episodic memory loss, a pattern that is consistent with Alzheimer’s Disease. In sum we found a dichotomic distinction between brain regions affected by developmental or ageing changes in Down syndrome.

## Teaser

Adults with Down syndrome show atypical brain morphology from altered neurodevelopment and pathological ageing. Using voxel-based morphometry, Jorge *et al*. identified age-related brain changes distinct from neurodevelopmental anomalies, the former being primarily occurring in regions vulnerable to Alzheimer’s disease. Age-related regional patterns of atrophy correlate with episodic memory decline.

## Graphical Abstract

**Graphical Abstract:**
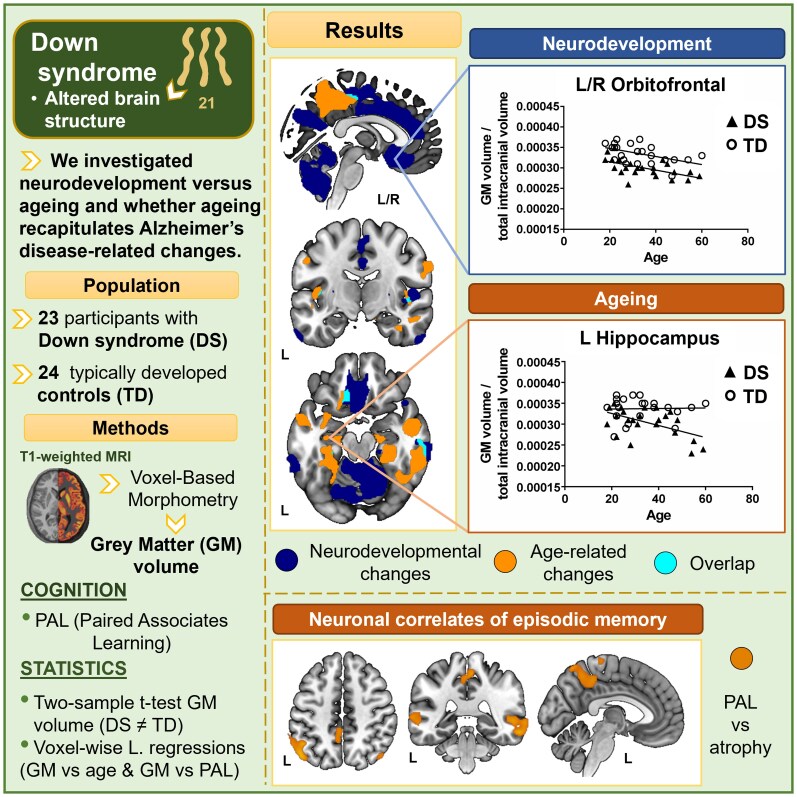

## Introduction

Individuals with Down syndrome (DS) exhibit significant neurodevelopmental brain abnormalities and face heightened risk of early-onset Alzheimer’s disease (AD), driven by the triplication of chromosome 21.^1,2^ Disentangling early neurodevelopmental alterations from the late age-related neurodegenerative processes is critical for understanding the distinct neurobiological mechanisms at play and for identifying potential biomarkers capable of detecting AD pathology at its earliest stages in individuals with DS.

According to previous studies, the neuroanatomy of DS is characterized by smaller brain sizes and reduced global grey matter (GM) volumes compared with typically developed individuals.^3-7^ At the regional level, significant GM volume reductions were consistently observed in the cerebellum^8-12^ and frontal lobe,^9,12^ while the parietal cortices tended to be found relatively preserved.^5,9,13^ Conversely, increases in GM volume were previously noted in the parahippocampal gyrus,^10,14^ putamen,^4,8,15^ and thalamus.^5,13^ Some studies have reported specific hippocampal subfields reductions.^12,16^ Additionally, early brain atrophy occurs in DS due to lifelong excessive accumulation of amyloid-β (Aβ) pathology, resulting from triplication of the gene encoding APP (amyloid precursor protein).^17-20^ This condition may therefore be defined as a genetically determined form of AD.^1,21^ In accordance, a wealth of studies has verified that by their 40s, nearly all individuals with DS exhibit the characteristic neuropathological hallmarks of AD, including abnormal Aβ deposits, neurofibrillary tangles and brain atrophy in overlapping brain systems.^2,22,23^ During the fourth decade of life, subtle declines in episodic memory,^24^ critical for learning and recalling spatiotemporal information, may also start to manifest, signalling progression from preclinical to prodromal stages of AD.^24-30^ After this age, there is a very high risk of developing further AD-related cognitive impairment,^24,31,32^ with dementia diagnosis commonly occurring in individuals over 50 years old.^33-35^

The strong age dependence of AD-related neuropathology in DS underscores the need to examine whether neurodegeneration in DS mirrors the AD pattern seen in the general population or reflects any DS-specific ageing traits. Elucidating age-related structural brain changes in DS is essential for identifying robust biomarkers that can facilitate early diagnosis, monitor disease progression, and inform the design of targeted clinical trials. Furthermore, such investigations may provide valuable insights into the fundamental mechanisms underlying early AD neuropathology. However, existing studies in DS are scarce^11,15,36-42^ and limited by region of interest (ROI)-based approaches, which introduce spatial bias, and by the relative lack of comparisons with neurotypical individuals, hindering the distinction between neurodevelopmental changes and those driven by ageing.

Thus, the main objectives of the our study were to: (i) to separate effects specific to neurodevelopment from those associated with ageing, including AD-related changes and DS-specific changes; and (ii) to investigate in DS individuals whether brain anatomy was significantly related to episodic visual memory decline, as measured by Paired Associates Learning (PAL)^43-46^—a test sensitive to AD progression at predementia stages in DS.^24,28^ To achieve this, we firstly compared brain GM volume maps, obtained through voxel-based morphometry (VBM) between DS individuals over a sufficiently large age range (18–59 years) and typically developed (TD) individuals, matched for age and sex. Subsequently, we investigated in DS group the effects of age on cortical GM by comparing it to TD, as well as the relationship between these and PAL performance.

Given the lack of studies comparing structural data between DS at preclinical/prodromal stages of AD with TD during using VBM, we asked whether neurodevelopmental changes in DS are independent from age-related effects on brain integrity, and into what extent these age-related changes fully recapitulate AD-related and cognitive changes.

## Materials and methods

### Participants

We recruited 30 adults with DS (9 females, 18–59 years) through services for individuals with intellectual disabilities in Portugal and 24 age- and sex-matched TD controls (8 females, 18–60 years) from the local community. TD participants were screened for neurodevelopmental, neurological, and psychiatric disorders and MRI contraindications. For DS participants, caregivers completed a semi-structured interview to identify medical conditions that could interfere with the study. Exclusion criteria included other neurological disorders, acquired CNS disorders, traumatic brain injury, or psychiatric illness. Eligible DS participants underwent ophthalmologic screening to rule out severe visual impairment and completed a neuropsychological battery in two sessions. All participants underwent MRI scanning at ICNAS (University of Coimbra). The study was approved by the Faculty of Medicine Ethics Committee (CE-058/2019) and conducted in accordance with the Declaration of Helsinki. Written informed consent and/or assent were obtained from all participants and their legal guardians.

### Cognitive assessment

DS participants completed a comprehensive neuropsychological battery assessing global cognition, memory, executive function, attention, processing speed, and sensorimotor skills. General cognitive ability (Intelligence Quotient) was measured using the Portuguese version of the Wechsler Adult Intelligence Scale-Third Edition (WAIS-III),^47,48^ see Breia *et al*.^49^ for more information. Specific domains were assessed with CANTAB,^43^ a validated computerized battery for adults with DS.^43,46,50,51^ Selected CANTAB tasks are widely used in DS research^46,52-54^ and are sensitive to age-related decline.^24,28,50,55-58^ Specifically, we used PAL for accessing episodic memory function, Pattern Recognition Memory (PRM) for visual pattern recognition memory, Multitasking Test (MTT) for executive functioning, Motor Screening Task (MOT) for sensorimotor functioning and reaction time (RTI) was used to access motor and mental response speeds (see Supplementary Table 1 for detailed information). We also included a verbal fluency (VF) test (food and animal categories) as an additional executive function measure.^59-61^ Dementia symptoms were screened using the Dementia Screening Questionnaire for Individuals with Intellectual Disabilities (DSQIID),^62,63^ completed by caregivers. The DSQIID was adapted to the Portuguese language through a rigorous translation process after obtaining formal authorization and content validation from the original authors. The cognitive evaluation was conducted across two visits scheduled < 1 month apart. The first visit included the WAIS-III, MOT, and PAL, while the second visit consisted of the PRM, RTI, and MTT tasks, as well as the VF test.

### MRI acquisitions

Structural MRI was acquired on a Siemens MAGNETOM Prisma fit 3T scanner with a 20-channel head coil. High-resolution T1-weighted MP2RAGE (Magnetization Prepared 2 Rapid Gradient Echo) images were obtained (TR/TE/TI1/TI2 = 5000/3.11/1100/700 ms; flip angles = 2°/5°; voxel size = 1 mm^3^; FOV = 256 mm^2^; TA = 10 min 35 s) to improve GM/WM (white matter) contrast. A T2-weighted FLAIR (Fluid-Attenuated Inversion Recovery) sequence was also acquired to detect hyperintense lesions (TR/TE/TI = 8000/83/2500 ms; voxel size = 0.7 × 0.7 × 3 mm^3^; a FOV = 191 × 235 mm^2^; TA = 3 min 28 s). As a first quality control step to ensure the reliability of the anatomical data, all MRI scans were acquired under direct supervision to minimize motion, and sequences were repeated when necessary. Prior to pre-processing, raw T1-weighted images were visually inspected for motion artefacts, and the FLAIR sequence was reviewed to confirm the absence of lesions that could interfere with segmentation or normalization.

#### MRI pre-processing and VBM-GM measurements

VBM was performed using the Computational Anatomy Toolbox (CAT12.9) (version 2560) within SPM12 (Wellcome Trust Centre for Neuroimaging, London, UK) in MATLAB (version R2020a). Due to MP2RAGE background noise, images were first denoised using BrainVoyager’s (version 22.4) MP2RAGE Background Denoising tool. All the next voxel-based processing steps were automatically conducted on CAT12 as described in detail elsewhere^64,65^ using the default settings. In short, the anatomical images were initially denoised with a spatially adaptive non-local means (SANLM) filter^66^ and corrected for intensity inhomogeneities. Then SPM’s standard unified segmentation^67^ was applied for the skull stripping and initial tissue segmentation using Tissue Probability Maps (TPMs). WM hyperintensities were spotted (to be considered later during the spatial registration), and local intensity transformation was performed to minimize the effects of higher GM intensities. For the final tissue segmentation into GM, WM, and CSF an adaptive maximum a posteriori (AMAP) segmentation was applied.^68^ The spatial normalization was achieved by aligning each subject’s brain to a standard MNI (Montreal Neurological Institute) template using the Geodesic Shooting.^69^ To account for changes in volume resulting from the registration process, adjustments were made using Jacobian modulation.^70^ As a second quality control step, all segmentation and normalization outputs underwent detailed visual inspection. CAT12’s Image Quality Rating (IQR), sample homogeneity metrics, and quartic mean *Z*-score values were examined to identify potential outliers.

We then estimated for each participant the total intracranial volume (TIV) used in the subsequent statistical analyses to adjust for the global individual differences in head size. Lastly, VBM-GM volume outputs were smoothed with an 8 mm FWHM (Full width at half maximum) Gaussian filter. Additionally, to assess whether the expected a priori decreases in hippocampal subfields GM could be detected, one subsequent sensitivity analysis was performed using a 6 mm FWHM Gaussian filter.

#### Voxel-based approach: cluster-defining thresholds, extent criteria, and mask preparation

Voxel-wise analyses were performed using CAT12/SPM12 statistical models. Whole-brain maps were generated using an initial uncorrected voxel-wise threshold. These were selected to ensure anatomical specificity and produce clear, interpretable clusters for subsequent ROI analyses. Significant clusters were identified using false discovery rate (FDR)-corrected *q*-values at the cluster-level or peak-level, as appropriate. No correction was applied in the exploratory analysis.

Binary masks of significant clusters were exported from CAT12, and mean GM volumes were extracted using an in-house script and normalized by each participant’s TIV (mean volume/TIV). These TIV-adjusted values were then used for ROI-based linear regressions and slope analyses. Additional masks of the hippocampal subfields were also obtained using the CoBrA atlas implemented in CAT12,^71^ which is publicly available at https://www.cobralab.ca/hippocampus-subfields. This atlas provides separate labels for CA1 (cornu ammonis), CA2/CA3, CA4/dentate gyrus, the subiculum and the stratum radiatum/stratum lacunosum/stratum moleculare (SR/SL/SM).

We also conducted a sensitivity analysis of the hippocampus and its subfields using a 6 mm smoothing FWHM Gaussian filter, reprocessing the VBM pipeline accordingly.

### Statistical analysis

#### Demographic and cognitive data analysis

Between-group age differences were tested with a two-sample *t*-test, and sex differences with Fisher’s Exact Test. Associations between neuropsychological scores and age were examined using Pearson correlations. These analyses were performed in SPSS (version 22) with statistical significance set at *P* < 0.05.

#### Group differences in GM volume maps and regional age-related trajectories

Between-group comparisons of whole-brain VBM-GM maps were performed using two-sample *t*-tests. Although TIV was initially included as a covariate, its strong correlation with GM measures led us to adopt global scaling (i.e. proportional scaling of each GM image by that participant’s TIV) to preserve variance.^72^ This approach was applied consistently across all statistical modules. Significant clusters were identified using a voxel-level threshold of *P* < 0.001 (uncorrected), a cluster-extent threshold of 150 voxels, and a subsequent peak-level FDR-correction at *q* ≤ 0.05. Peak-level correction was preferred in this analysis to retain as significant effects in the hippocampus and parahippocampal gyrus, regions well documented as being neuroanatomically altered in individuals with DS.

To distinguish neurodevelopmental differences from age-related decline, we examined age trajectories within regions showing reduced GM volume in DS (hereafter referred to as DS-reduced regions). Linear regressions of TIV-normalized mean GM volume on age were computed for each reduced region, separately for each group. Group differences in GM-versus-age slopes were assessed in GraphPad Prism (version 6.01) using a significance threshold of *P* < 0.05 (uncorrected for multiple comparisons). The software makes this evaluation by testing the null hypothesis that the slopes are identical (equivalent to an Analysis of Covariance approach), following the method presented in Zar *et al*.^73^ as described in detail in the software documentation page.^74^ The slope patterns provide the following interpretative framework for regression analyses in DS-reduced regions: same intercept and different age-related slope (defining specific age-related differences), different intercept (implies initial neurodevelopmental differences) and same slope (indicates no age-related differences).

Given prior evidence of neurodevelopmental reductions in specific hippocampal subfields in DS, we applied the same regression and slope-comparison procedures to anatomically defined hippocampal subfield masks from the CoBrA atlas.^75^ We also conducted a hippocampus-focused sensitivity analysis by rerunning the VBM pipeline with a smaller 6 mm FWHM Gaussian smoothing kernel, following the procedure of a previous study.^12^

#### Discriminating neurodevelopmental and age-related GM volume changes in participants with DS

Whole-brain voxel-wise linear regressions of age on GM maps were performed to investigate age-related GM changes separately in the group of participants with DS and TD group. The effects of age on GM volume were examined within each group using an initial voxel-level threshold of *P* < 0.0001 (uncorrected) and a cluster-extent threshold of 150 voxels, followed by cluster-level FDR correction at *q* ≤ 0.05. For this analysis a stricter voxel-level threshold was required to prevent large, merged clusters and to enable regional differentiation.

Mean GM volumes from clusters showing significant age-related GM reductions in DS were extracted for all participants (DS and TD). Linear regressions of TIV-normalized mean GM volume on age were computed separately for each group, and slope comparisons were performed to determine whether age-related reductions in DS differed statistically from those in the TD group. An FDR-corrected significance threshold of *q* ≤ 0.05 was applied to account for multiple comparisons. Statistically significant slope differences were interpreted as evidence of group-specific age-related GM changes, whereas non-significant slope differences indicated similar age-related trajectories between groups.

We then assessed the overlap between neurodevelopmental and age-related GM changes in the group of individuals with DS to determine whether these processes affect distinct or shared brain regions. Overlap was quantified using Dice coefficients.

#### Association between cognitive performance and GM volume in participants with DS

Whole-brain voxel-wise linear regressions of GM volume on PAL first-attempt memory scores (CANTAB) were performed to investigate the association between GM volume and episodic memory decline in the DS group. This exploratory analysis used a voxel-level threshold of *P* < 0.01 (uncorrected) and a conservative cluster-extent threshold of 320 voxels, corresponding to the expected minimal detectable cluster size for this design.

## Results

### Demographical and cognitive data

Of the 30 recruited DS participants, one declined and two did not meet inclusion criteria. One completed only the cognitive assessment, and 26 completed both the core neuropsychological battery and MRI. Two participants (one TD and one DS) were flagged as potential outliers due to quartic mean *Z*-scores exceeding 2 standard deviations (TD: 1.710; DS: 1.526). As noted in CAT12 documentation, such values do not require automatic exclusion but warrant careful inspection.^72^ After careful visual review, neither dataset showed major artefacts or segmentation problems, and both were retained in the final sample. Three MRI datasets were excluded for motion artefacts (ages 19, 23, and 56), resulting in 23 DS and 24 TD participants for analyses requiring both imaging and cognitive data. Analyses based solely on cognitive measures included up to 27 DS participants.

Participants whose MRI scans were excluded did not attend the second cognitive evaluation visit, resulting in missing cognitive data. Because not all participants completed every test, sample sizes vary across cognitive measures. A CONSORT-style flow diagram summarizing recruitment, exclusions, and final sample sizes is provided in Supplementary Fig. 1. The demographic analyses revealed that the group with DS and the TD group were well matched for age and gender distribution. Results of the statistical comparisons along with the demographic characteristics of both groups are presented in Supplementary Table 2. The age distribution of the final DS sample included in the imaging and imaging-cognitive analyses are shown in Supplementary Fig. 2. A complete set of neuropsychological test scores for participants with DS, along with their associations with age, is presented in Supplementary Table 3. DS participants scored a mean IQ of 53.18 ± 5.04 (range: 45–66) on WAIS-III, classified as ‘extremely low’ per Wechsler and consistent with DSM-5-TR [Diagnostic and Statistical Manual of Mental Disorders (Fifth Edition)]^76^ criteria for intellectual disability. Age negatively impacted PAL first attempt memory score and increased MOT response times; RTI showed a trend toward slower responses with age. No significant age effects were found for VF, MTT, or PRM. DSQIID screening identified only one participant with possible dementia onset (score = 30) confirming the sample represents virtually preclinical/prodromal AD stages. Biomarker confirmation was limited to ^11^C-PiB PET, available for 21 participants and read by an experienced nuclear medicine physician. Only five participants over 40 years old were Aβ-positive.

### Group differences in GM volume maps

**Figure 1:**
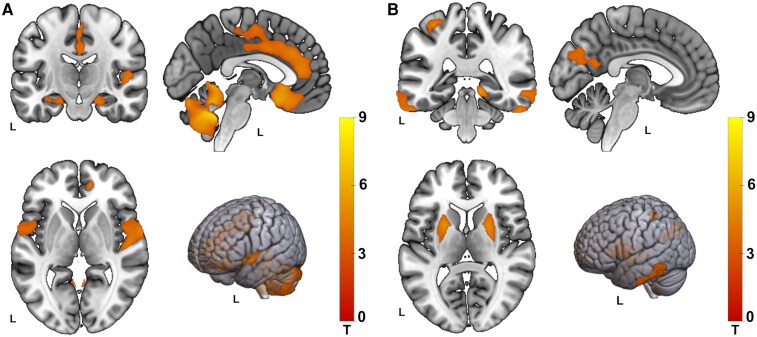
Group differences in GM volume across the whole brain. (**A**) Regions showing significantly reduced volume in the DS group (*n* = 23) relative to the TD group (*n* = 24) (DS < TD). (**B**) Regions showing significantly increased volume in the DS group relative to the TD group (DS > TD). Statistical maps were obtained using a two-sample *t*-test with an initial voxel-wise threshold of *P* < 0.001 (uncorrected) and a cluster-extent threshold of ≥150 voxels, followed by peak-level FDR correction (*q* ≤ 0.05), TIV was inserted with global scaling. DS, Down syndrome; GM, grey matter; L, left; *n*, number of participants; T, T-statistic; TD, typically developed; TIV, total intracranial volume.

Whole-brain VBM-GM results are summarized in Table 1 and illustrated in Fig. 1. After TIV scaling, DS participants showed significant bilateral GM reductions in the cerebellum, temporal lobe (including superior temporal gyrus, insula, and transverse temporal gyrus), orbitofrontal cortex, subgenual area, and a large cluster spanning anterior/middle cingulate and anterior prefrontal cortex (PFC). Additional reductions were observed in the left hippocampus, with trends in the right hippocampus. Conversely, DS participants exhibited GM volume increases in bilateral cuneus/precuneus, inferior and middle temporal gyri, putamen, and enlargement of the left postcentral and right parahippocampal gyri.

**Table 1:** Regions evidencing statistically significant differences in GM volume between the group with DS and TD group

Brain region	Side	Cluster-level		Peak voxel-level						
		Cluster size	qFDR^a^	MNI coordinates			T	Equi-Z	qFDR^b^	*P* ^c^
				x	y	z				
**DS < TD**										
**Cerebellum**	L/R	27 008	<0.001	0	−50	−33	8.52	6.54	<0.001	<0.001
**Temporal (BA 22/13)**	R	2466	0.002	56	14	−8	6.54	5.45	0.003	<0.001
**Orbitofrontal BA (11/25)**	L/R	2894	0.001	−9	38	−18	5.96	5.09	0.006	<0.001
**Anterior cingulate cortex (BA 10/23)**	R/L	5422	<0.001	12	44	8	5.78	4.97	0.008	<0.001
**Temporal (BA 22/13)**	L	1001	0.067	−56	9	−5	5.54	4.81	0.011	<0.001
**Hippocampus**	L	181	0.66	−18	−20	−18	4.75	4.25	0.045	<0.001
**Hippocampus**	R	170	0.66	20	−20	−15	4.51	4.07	0.076^d^	<0.001
**DS > TD**										
**Cuneus/precuneus (BA 18/19/7)**	L/R	1869	0.020	−17	−71	39	7.40	5.95	0.001	<0.001
**Inferior/middle temporal gyrus (BA20/21)**	R	1843	0.020	60	−29	−30	6.06	5.15	0.016	<0.001
**Inferior/middle temporal gyrus (BA20/21)**	L	1688	0.020	−57	−32	−30	5.71	4.93	0.029	<0.001
**Putamen**	L	1020	0.073	−21	3	11	5.52	4.80	0.038	<0.001
**Parahippocampal gyrus (BA36)**	R	253	0.53	15	−35	−11	5.38	4.70	0.043	<0.001
**Putamen**	R	1452	0.028	23	0	12	5.32	4.66	0.043	<0.001
**Postcentral gyrus (BA 1)**	L	417	0.39	−32	−36	56	5.28	4.63	0.043	<0.001

BA, Brodmann area; DS, Down syndrome; L, left; MNI, Montreal Neurological Institute space; *n*, number of participants; R, right; TD, typically developed.

Results of the between-group two-sample *t*-test analysis (DS group, *n* = 23; TD group, *n* = 24) obtained with an initial voxel-wise *P* < 0.001 uncorrected, cluster ≥150 voxels, followed by peak-level correction for multiple comparisons qFDR < 0.05. ^a^FDR-corrected cluster-level *q-*value. ^b^FDR-corrected peak-level *q-*value. ^c^Uncorrected peak-level *P*-value. ^d^Marginal significance.

### Between-group comparison of age-related structural patterns in overall DS-reduced regions

**Figure 2:**
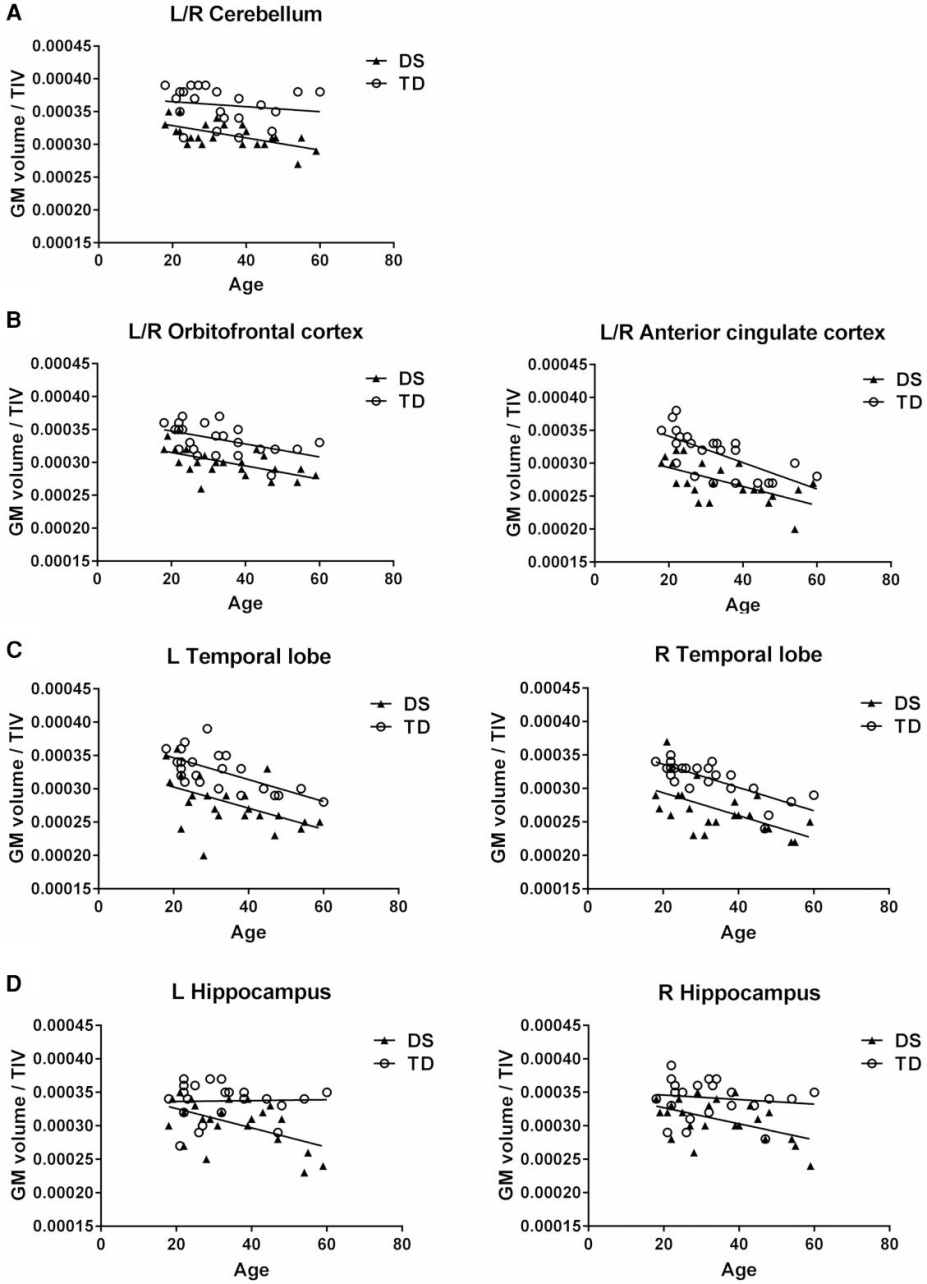
Relationship between regional brain measures and age in the DS and TD groups. The plot shows the linear regression of mean GM volume (proportional to TIV) as function of age in regions where the DS group (*n* = 23) demonstrates reduced GM volume compared to the TD group (*n* = 24) (DS < TD). Each point represents an individual participant’s mean GM volume within the selected regions of interest (proportional to TIV). For (**A**) the bilateral cerebellum, (**B**) the bilateral orbitofrontal and anterior cingulate cortices, and (**C**) the bilateral temporal regions, although regional GM volume is lower in the DS group, both groups show similar slopes, suggesting that age-related volume changes progress at comparable rates across most regions. Conversely, (**D**) Hippocampal volume appears similar to that of TD participants at younger ages but shows a steeper decline with age in the DS group, indicating more pronounced structural deterioration in older individuals. Statistics from the linear regressions are detailed in Supplementary Table 4. Values on the *y*-axes are normalized to TIV, which accounts for their magnitude and renders them unitless. DS, Down syndrome; GM, grey matter; L, left; *n*, number of participants; R, right; TD, typically developed; TIV, total intracranial volume.

Figure 2 shows the relationship between GM volumes and age in selected regions of interest. Most DS-reduced regions show lower GM volume but parallel age-related slopes, supporting a neurodevelopmental origin. The hippocampus is the exception: it appears preserved in early adulthood but shows a later, divergent age-related decline in DS, consistent with accelerated ageing in this region.

Comparisons of GM-versus-age regression slopes in DS-reduced regions revealed intercept differences without slope differences in the cerebellum, temporal regions, orbitofrontal cortex, and cingulate cortex, indicating predominantly neurodevelopmental structural differences. Results of the slopes comparison are displayed in Supplementary Table 4. In contrast, the hippocampus showed similar intercepts between groups but visually steeper age-related decline in DS (See Fig. 2). Formal slope comparisons confirmed significant age-related GM decline in the left hippocampus, in DS (i.e. the slope was statistically different from zero) (*F*(1,21) = 7.81, *P* = 0.011) and differed from TD slope (*F*(1,22) = 4.41, *P* = 0.042), suggesting age-driven structural reduction. The right hippocampus also declined significantly with age in DS (*F*(1,21) = 6.89, *P* = 0.016), unlike in TD (*F*(1,22) = 0.43, *P* = 0.52). However, the between-group slope difference for the right hippocampus was not significant (*F*(1,43) = 1.57, *P* = 0.22), aligning with marginal effects observed in broader comparisons. Thus, the right hippocampus shows an age-related decline in DS, although not significantly different from TD, potentially suggesting greater resilience or a later onset of age-related degeneration.

Given prior evidence reporting neurodevelopmental GM-volume differences in specific hippocampal subfields,^12,16^ we subsequently performed subfield-level analyses. The corresponding results are presented in Supplementary Fig. 3 and Supplementary Table 5. Between-group comparisons of the GM-versus-age slopes revealed that, bilaterally across all subfields examined, both groups showed similar values at younger ages (i.e. comparable intercepts). However, the age-related slopes differed significantly between groups bilaterally, indicating, consistent with our interpretative framework, that the observed differences are age-related.

The sensitivity analysis using a smaller smoothing kernel (6 mm FWHM) restricted to the hippocampus showed comparable intercepts but significant age-related slope differences in all subfields (except CA2/CA3), suggesting an age-related origin of the structural differences. Sensitivity-analysis results are presented in Supplementary Fig. 4 and Supplementary Table 6. Despite the absence of age-related effects in CA2/CA3, no neurodevelopmental differences are apparent in this subfield, as indicated by the similar intercepts observed between groups.

### Age-related effects on whole brain GM volume in the group with DS and TD group

**Figure 3:**
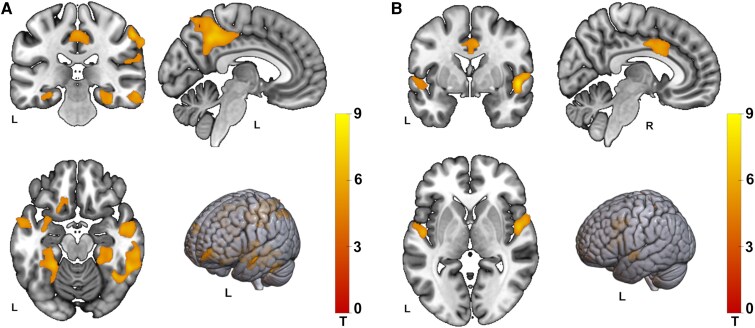
Regions evidencing a significant negative association between GM volume and age. (**A**) Regions in which GM volume decreases significantly with age in the DS group (*n* = 23). (**B**) Regions in which GM volume decreases significantly with age in the TD group (*n* = 24). Statistical maps were obtained using voxel-wise linear regression analysis of GM volume on age, with an initial threshold of *P* < 0.0001 (uncorrected) and a cluster-extent threshold of ≥150 voxels, followed by cluster-level FDR correction (*q* ≤ 0.05). TIV was inserted with global scaling. DS, Down syndrome; GM, grey matter; L, left; *n*, number of participants; R, right; T, T-statistic; TD, typically developed; TIV, total intracranial volume.

Age-related effects on brain structure differed between groups. Results are summarized in Supplementary Table 7 and illustrated in Fig. 3. Participants with DS showed widespread GM reductions, most pronounced in parietal and temporal lobes, whereas TD participants exhibited only subtle declines. In the group with DS, bilateral age-related reductions were observed in the angular and supramarginal gyri, precuneus, lingual and fusiform gyri, parahippocampal gyrus, and clusters extending to the hippocampus and cerebellum. The right temporal lobe showed diffuse reductions across superior, middle and inferior gyri, while the left temporal lobe displayed more restricted declines in the superior gyrus. Additional smaller clusters appeared in left orbitofrontal cortex, insula, anterior PFC, and right dorsolateral PFC (DLPFC). Bilateral age-GM associations were observed, with a more pronounced effect in the right hemisphere. In TD, significant age-related GM loss was limited to the superior temporal and cingulate gyri.

### Comparison of brain ageing in the group of individuals with DS to the TD group

We compared age-related GM reductions observed in individuals with DS to corresponding regions in TD. Results are shown in Table 2. Most cortical regions showing age-related decline in individuals with DS differed significantly from TD, indicating accelerated ageing in DS. Many differences remained significant after FDR correction, particularly in right temporal and parietal clusters, and in bilateral posterior cingulate cortex (PCC)/precuneus. This pattern suggests selective vulnerability of these regions to ageing in individuals with DS, likely reflecting both DS-specific and AD-related neuropathology.

**Table 2:** Group differences in clusters showing significant age-related volume decline in the group with DS

Region	Side	Group	Slope	Difference from zero	Slope differences between groups		
					*F*(1,43)	*P* ^a^	qFDR^b^
**1—Supramarginal gyrus/angular gyrus (BA 40/39)**	R	DS	−3.04e−006	<0.001	9.58	0.0035	**0**.**011**
		TD	−1.49e−006	<0.001			
**2—Fusiform gyrus/middle and inferior temporal gyrus (BA 37/21/20)/cerebellum**	R	DS	−2.76e−006	<0.001	20.04	<0.001	**0**.**0013**
		TD	−1.05e−006	0.0011			
**3—Superior and middle temporal gyrus)/angular gyrus (BA 22/21/39)**	R	DS	−2.67e−006	<0.001	6.93	0.012	**0**.**031**
		TD	−1.37e−006	0.0011			
**4—PCC/precuneus (BA 31/7)**	L/R	DS	−3.37e−006	<0.001	11.08	0.0018	**0**.**0078**
		TD	−1.45e−006	0.0012			
**5—Lingual and fusiform gyrus (BA 37)/cerebellum/hippocampus**	L	DS	−2.47e−006	<0.001	6.07	0.018	**0**.**033**
		TD	−1.19e−006	0.0057			
**6—Angular gyrus/supramarginal gyrus (BA 39/40)**	L	DS	−2.75e−006	<0.001	6.176	0.017	**0**.**033**
		TD	−1.11e−006	0.050			
**7—Superior and middle temporal gyrus (BA 22/21)**	L	DS	−2.50e−006	<0.001	3.95	0.053	0.069
		TD	−1.22e−006	0.018			
**8—Middle and superior temporal gyrus/fusiform gyrus/parahippocampal gyrus (BA 21/22/37/36)/hippocampus**	R	DS	−2.57e−006	<0.001	11.85	0.0013	**0**.**0078**
		TD	−1.03e−006	0.008			
**9—Inferior temporal gyrus (BA 20)/cerebellum**	L	DS	−2.46e−006	<0.001	1.55	0.22	0.22
		TD	−1.76e−006	<0.001			
**10—Orbitofrontal cortex (BA11)**	L	DS	−1.66e−006	<0.001	5.16	0.028	**0**.**046**
		TD	−5.80e−007	0.16			
**11—Insula (BA13)**	L	DS	−2.41e−006	<0.001	2.70	0.11	0.13
		TD	−1.53e−006	<0.001			
**12—Anterior PFC (BA10)**	L	DS	−2.57e−006	<0.001	4.86	0.033	**0**.**048**
		TD	−1.03e−006	0.085			
***13—DLPFC* (BA9)**	R	DS	−2.82e−006	<0.001	1.82	0.16	0.17
		TD	−1.87e−006	<0.001			

BA, Brodmann area; DLPFC, dorsolateral prefrontal cortex; DS, Down syndrome; L, left; *n*, number of participants; PCC, posterior cingulate cortex; PFC, prefrontal cortex; R, right; TD, typically developed.

Results of the between-group comparison (DS group, *n* = 23; TD group, *n* = 24) of regression slopes using an equivalent ANCOVA procedure. ^a^Uncorrected *P*-values and ^b^FDR-corrected *q*-values (Benjamini-Hochberg, *α* = 0.05) are shown. Values in bold are significant (*q* < 0.05).

### Overlap between neurodevelopmental and age-related structural changes

**Figure 4:**
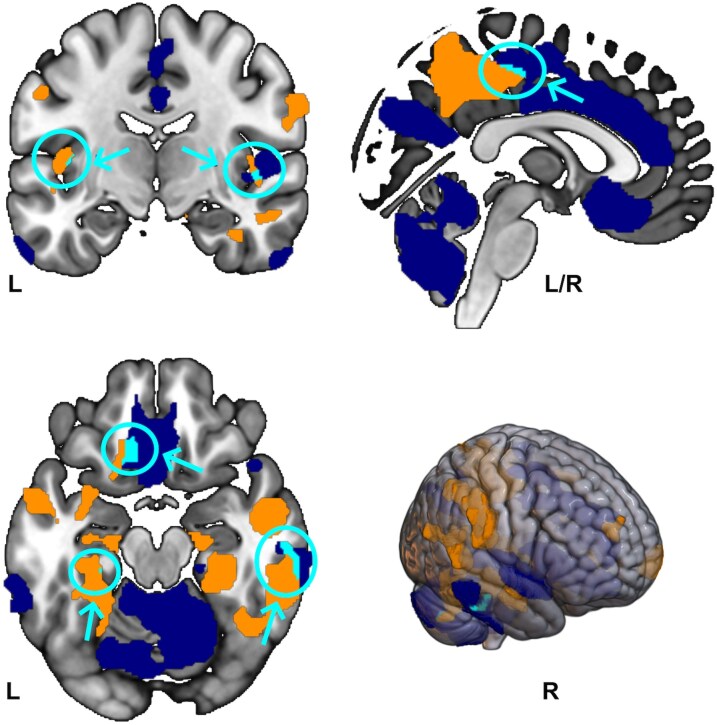
Overlap between GM volume neurodevelopmental and age-related changes in the DS group (*n* = 23). Dark blue indicates neurodevelopmental changes; orange indicates age-related changes; and sky blue (circled) denotes regions of overlap between the previous two. The overlap was quantified using Dice coefficients. DS, Down syndrome; GM, grey matter; L, left; *n*, number of participants; R, right.

Our results indicate minimal overlap between neuroanatomical and age-related changes in DS, as illustrated in Fig. 4. Small overlapping clusters were found mainly in the right inferior/middle temporal gyrus, bilateral PCC, bilateral insula, right cerebellum, left orbitofrontal cortex, and left parahippocampal gyrus. These regions included areas of both GM volume decrease and increase in DS, suggesting that, in general, neurodevelopmental alterations did not modify susceptibility to age-related neurodegeneration.

### Association between cognitive performance and GM volume in participants with DS

**Figure 5:**
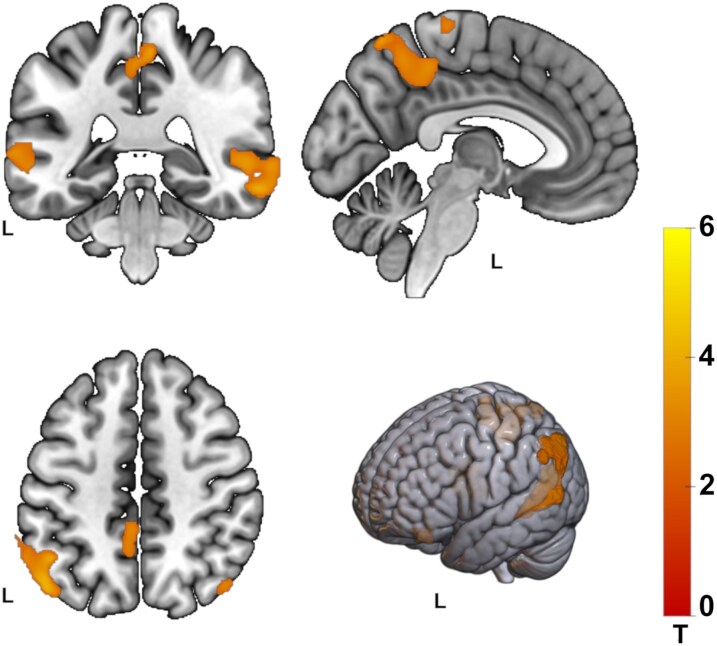
Regions evidencing a significant positive association between GM volume and PAL first memory score in the DS group (*n* = 23). Statistical maps were obtained using voxel-wise linear regression analysis of GM volume versus PAL score, with a threshold of *P* < 0.01 (uncorrected) and a cluster extent threshold of ≥320 voxels. TIV was inserted with global scaling. DS, Down syndrome; GM, grey matter; L, left; *n*, number of participants; PAL, paired associates learning; T, T-statistic; TIV, total intracranial volume.

The first attempt memory score on the PAL test showed significant positive correlations with GM volumes in bilateral angular gyrus (extending into supramarginal and middle temporal gyri on the left), right middle temporal gyrus, bilateral precuneus (extending to PCC and precentral gyrus), and left ventrolateral PFC (Fig. 5, Supplementary Table 8). This indicates reduced GM volume in these regions was associated with poorer episodic memory performance. Behaviour-GM volume associations involved both hemispheres, with slightly greater involvement on the left.

### Imaging endpoints and sample size considerations for DS trials

To evaluate the potential of GM hubs as imaging endpoints in DS clinical trials, we conducted an exploratory analysis using the regression coefficients derived from the regions showing significant age-related GM decline, particularly the PCC/precuneus (cluster number 4 from Table 2). In this cross-sectional cohort, the age-GM volume regression coefficients β (slope) show that the age-associated decline in PCC/precuneus GM volumes was significantly higher in the DS group (*β*_DS_ = −3.37 × 10^−6^/year) compared with the TD group (*β*_TD_ = −1.45 × 10^−6^/year). This represents an accelerated atrophy rate of Δ*β*_TD_,_DS_ = 1.926 × 10^−6^/year in the DS group. This differential rate of neurodegeneration provides a clear target for disease-modifying interventions aimed at slowing GM loss in DS toward normative trajectories.

To demonstrate the practical utility of these parameters for clinical trial design in DS, we utilized the observed cross-sectional uncertainty of the slope estimates to estimate sample size requirements of a longitudinal study. Assuming a two-sided *α* = 0.05 and 80% power, detecting a 30% reduction in the rate of DS-associated GM volume decline (Δ*β*_DS_,_DS_ = 1.012 × 10^−6^/year) would require ∼60 participants per arm in a 1-year, two-arm parallel-group trial (active treatment versus placebo). Given the cross-sectional nature of these associations, the power calculations remain exploratory and should be interpreted with caution. Future longitudinal studies are necessary to more accurately define within-subject trajectories of decline and their underlying variability.

## Discussion

The current study sought to distinguish neurodevelopmental structural alterations in DS from those related to ageing and early-stage AD-like changes in relation to cognitive performance. As a strategy, we first compared brain anatomy between DS individuals and age and sex-matched TD and then assessed patterns of brain ageing across groups. DS exhibited a dual pattern of both significantly smaller and larger regional GM volumes relative to TD, despite having an overall smaller brain size. In addition, DS was associated with precocious structural brain ageing with a spatial pattern broadly resembling that observed in prodromal AD.

Compared with the TD, individuals with DS showed significant reductions in GM volume bilaterally in the cerebellum, superior temporal gyrus, orbitofrontal cortex, cingulate gyrus, and left hippocampus. These results are broadly coherent with previous volumetric work, particularly in non-demented DS subjects.^12,16,37,38,40,77^ Given the expected age-related GM loss in our DS cohort^37,42,78^ (young to middle-aged adults), we examined whether GM reductions in these regions were attributable to neurodevelopmental factors (intercept differences) or later atrophic processes (age related reduction shown by increased slopes). Our results suggest that while neurodevelopmental factors likely explain structural differences in most regions, this was not the case for the hippocampus, where similar early-age profiles (same intercept) diverged significantly with advancing age (distinct regression sloped). Intriguingly, in spite of the existing literature suggesting that a smaller hippocampus is part of the DS neurodevelopmental phenotype,^9,10,79-81^ our results rather suggest that age-related mechanisms possibly account for its gross structural shrinkage, as the divergence of GM between groups found in this region appears to be driven mainly by older DS individuals. Consistent with this, prior studies in DS have suggested that hippocampal atrophy worsens near dementia onset^11,82-84^ and Aβ load.^15,85^ Notably, White *et al*.^12^ initially found a regional pattern of GM abnormalities in DS, very similar to what we attribute here to neurodevelopment, including no hippocampal volume changes. However, after reanalysis with a smaller smoothing kernel, they detected subtle differences in C3/C4 dentate hippocampal subregions. Similarly, Koenig *et al*.^16^ using high-resolution MRI, reported selective GM volume reductions in bilateral hippocampal subfields in young adults with DS. Conversely, our subfield analyses did not reveal clear neurodevelopmental differences within the hippocampal subfields; instead, they point toward clearcut and progressive age-related alterations. However, we acknowledge that VBM has limited sensitivity for detecting changes at the level of hippocampal subfields^86-88^ and that our sample may not include enough younger participants for such differences to be detected. Thus, subtle neurodevelopmental differences may exist in specific hippocampal subfields, whereas gross structural changes become more evident with increasing age. Overall, our findings support the use of global hippocampal volumes as a viable intergroup biomarker for AD pathology in DS, similar to what is observed in both sporadic and familial AD individuals.^89,90^

Conversely, in DS group significantly higher GM volumes were identified bilaterally in the cuneus, inferior/middle temporal gyrus and putamen, right parahippocampal and left postcentral gyri, compared with TD. Although parahippocampal gyrus and putamen GM augmentations have been widely reported in DS in previous neuroimaging studies,^4,8,10,12,37,38^ findings of GM increases in other cortical regions have been less constant among studies. Nonetheless, some studies have reported either preserved or augmented volumes in the parietal, temporal, and even frontal lobes after making statistical adjustments for total brain volume (inserting TIV as a covariate).^4,5,9,12,13,37,91^ Here, by controlling for differences in brain sizes with global scaling, we may have detected additional regional GM increases in the context of a smaller brain size in DS. Despite some inconsistencies with prior literature, a similar pattern of cortical GM preservation/increase suggests that non-demented DS individuals can be differentiated from neurotypical controls,^12,92,93^ and these enlargements have also been observed in DS children and adolescents,^4,5,9,81^ reinforcing the notion that volumetric enlargement of certain cortical regions is likely part of the DS neurodevelopmental phenotype and persists into adulthood.

The effects of ageing on whole brain anatomy were distinct across groups. In TD individuals, a significant interplay between GM integrity loss and age was denoted bilaterally in the superior temporal and middle cingulate gyri, consistent with previous findings in non-elderly adults.^94^ In contrast, the DS group exhibited a widespread pattern of age-related regional atrophy. The most significant reductions were denoted bilaterally in the supramarginal and angular gyri, precuneus, middle and inferior temporal gyri, fusiform gyrus, encompassing also part of the hippocampus and cerebellum, a finding that is consistent with previous work.^37,40,42,95^ Moreover, these age-related reductions in DS were statistically different from those in TD, supporting an atypical and accelerated brain ageing process in DS, with pronounced involvement of the right lateral and medial temporal cortex, inferior parietal lobe, and bilateral precuneus and PCC. Right-sided GM reductions were also only partially replicated in the left hemisphere; since, they were less extensive (smaller clusters), and some did not survive correction for multiple comparisons. This finding may indicate an overall higher susceptibility of the right hemisphere to the initial effects of ageing in DS, which is consistent with previous work in DS,^15,96^ and possibly represents a DS-specific ageing trait, as prior research has posited greater left hemisphere atrophy early in AD and healthy ageing.^97-99^ This posterior-dominant GM decline, involving medial posterior cingulate, precuneus, and lateral parieto-temporal cortices, closely mirrors patterns seen in preclinical and prodromal stages of both familial^100-106^ and sporadic AD,^107-111^ supporting its link to AD-related neuropathology in DS.^15^ Particularly, the precuneus and PCC, known hotspots and early targets of AD neuropathology^112,113^ and critical for episodic memory,^114,115^ showed significant bilateral shrinkage, suggesting their potential as reliable biomarkers of early AD also in DS.

Although the whole-brain VBM analysis provided early indications of hippocampal involvement, age-related effects were more clearly delineated in the ROI-based analyses. This pattern aligns with the enhanced sensitivity of ROI-based methods and is consistent with previous findings. For example, Teipel *et al*.^42^ reported that whole-brain VBM did not detect age-related hippocampal changes in individuals with DS, whereas their earlier study, using manual ROI-based volumetry on part of the same sample, successfully identified such age-related reductions.^40^ Overall, hippocampal atrophy appears more pronounced near dementia onset in both DS^11,15,82,84,116^ and AD,^89^ whereas early neurodegeneration predominantly affects a dominant posterior pattern, namely medial posterior cingulate, precuneus, and lateral parieto-temporal cortices, which are known earlier AD targets.^100-111^

Although APP gene triplication likely plays a key role in abnormal brain ageing in DS, over dosage of other chromosome 21 genes linked to ageing mechanisms may also contribute to premature brain atrophy,^117-120^ including atrophy in regions not typically involved preclinically in AD, such as the superior temporal gyrus, orbitofrontal cortex, and anterior PFC. Overall, current results align with recent findings by Kennedy *et al*.^96^ who reported that while DS individuals exhibited overlap mainly in the lateral and medial parietal lobes with familial AD, they also showed more diffuse cortical thinning on average. Furthermore, although it has been suggested that the regions evidencing neurodevelopmental changes might also be the ones subjected to enhanced ageing,^37,121^ the limited overlap observed here rather suggests that these phenomena are relatively independent.

Regarding cognitive function data, our analysis of the DS cohort revealed significant age-related declines in tests of episodic memory (PAL) and sensorimotor functions (MOT), whereas a tendency was observed to the processing speed and attention (RTI), consistent with previous literature^24,28-30^ and stressing their relevance as early cognitive indicators of AD neuropathology in individuals with DS. In contrast, no significant age-related associations were observed for the VF or MTT, suggesting that alterations in executive functioning appear to manifest at a later stage of cognitive decline, in concurrence with the findings of previous studies.^24,25,29,122,123^ Our outcomes are in line with previous studies resorting to the same battery.^49,124,125^ Fewer participants completed the MTT task, consistent with known increased difficulties in executive abilities in DS.^46,50,54,126^ On its turn, IQ showed a positive correlation with age, with older participants exhibiting higher scores. This pattern is consistent with evidence that cognitive development in individuals with DS can continue into mid adulthood.^127^ These findings suggest that intellectual disability severity did not drive age-related decline in other tasks.

The classification of these individuals as being in the preclinical or prodromal AD stages aligns with the well-established age dependent progression of AD pathology in DS.^22,23,128^ Given the nearly universal lifetime risk associated with APP overexpression, amyloid and tau pathology typically emerge decades before symptoms, supporting the view that individuals with DS may be considered preclinical even before biomarker positivity. Subtle episodic memory decline indicates the prodromal stage^24^ while broader impairment (as assessed with the DSQIID) reflects dementia. DSQIID screening identified only one participant with possible dementia onset (score = 30).

We further examined whether brain atrophy accounted for the decline specifically on PAL performance, as assessed by the first attempt memory score, because of its proven sensitivity to preclinical AD-related cognitive decline.^24,28^ Our results indicate that age-related reductions in PAL performance were associated with greater GM atrophy, particularly in the left angular gyrus (extending to the superior temporal gyrus), right middle temporal gyrus, precuneus bilaterally (extending to PCC), right frontal eye fields, and left ventrolateral PFC. This set of regions is known to subserve several cognitive processes underpinning episodic visual memory,^129-137^ and is broadly consistent with the findings of previous studies using other metrics of episodic memory and in larger cohorts of DS.^42,122^ Thus, the current study offers new insights into the neuroanatomical basis of performance deficits in the PAL task, likely associated with AD neuropathology in individuals with DS at predementia stages. Preferential associations between structural atrophy and PAL decline in AD-related brain hubs (bilateral precuneus and PCC), support its link to AD-related neuropathology in DS, reinforcing their potential use as reliable biomarkers of early AD in DS. Furthermore, this pattern of associations involving mainly PCC, precuneus, angular gyrus and lateral temporal regions, critical to the attentional and visuospatial processes underlying memory, may suggest subtle functional impairments of the networks and upstream processes of memory encoding rather than ‘pure focal memory impairment’ (disruption in the process of encoding and new memory formation) at predementia stages, which was already suggested to AD in the general population.^133,138^

### Limitations

This study has several limitations that should be acknowledged. First, the gender imbalance, with fewer than one third of the DS cohort being female may limit generalizability, although MRI studies in DS have not reported consistent neuroanatomical sex differences.^12,139^ Similarly, sex differences in brain ageing and AD-related changes in DS appear subtle and inconsistent compared with the general population.^52,140^ Although males may show earlier mid-life memory decline, core AD biomarkers (Aβ, tau, neurodegeneration) show minimal sex effects. Second, although our age distribution spans the adult lifespan, the limited number of younger DS participants may reduce the power to detect subtle neurodevelopmental differences, namely in hippocampal subfields. Accordingly, our finding of no neurodevelopmental differences in hippocampal GM volume should be interpreted with caution, and larger cohorts will be needed to confirm these results. Moreover, hippocampal subfield findings should be interpreted carefully given the known limitations of VBM’s sensitivity to hippocampal subfields.^88^

Third, volumetric accuracy can be uncertain when applying standard pipelines to cohorts with atypical brain morphology. We did not use DS-specific templates for segmentation and normalization, like much of the recent DS neuroanatomy research.^4,15,16,141^ Prior VBM studies that created DS or study specific templates reported neurodevelopmental and age-related patterns broadly comparable to ours,^12,42^ supporting the validity of standard workflows in DS. Moreover, when motion is minimal, analyses using DS-specific versus standard SPM templates produce similar results, suggesting that high quality, motion free data mitigates template related bias.^142,143^ We applied strict motion checks and excluded artefactual scans, which likely improved segmentation robustness. Additionally, reports on reduced GM/WM contrast in DS are mixed.^141,144^ To minimize related biases, we used high-resolution MP2RAGE, which improves tissue contrast and segmentation stability. Even so, residual contrast differences cannot be ruled out, and future work should explicitly control for potential GM/WM contrast effects.

Forth, given the absence of full biomarker confirmation (although we had information on Aβ imaging status consistent with prior reports^128^), PET-based Aβ measures alone are insufficient to definitively ascertain the stage of AD across the cohort. Moreover, we cannot guarantee that all individuals in the DS group exhibited AD-related pathology, which may introduce biological heterogeneity and limit the generalizability of our findings.

Finally, the cross-sectional design limits our ability to infer true trajectories of brain ageing. Group derived age slopes in a cross-sectional study cannot substitute for longitudinal trajectories. We propose futures studies should repeat MRI assessments at 18–36 months to quantify regional atrophy rates (e.g. PCC/precuneus and other DS AD hubs) and to test whether structural decline parallels changes in episodic memory and other cognitive functions. Such longitudinal data will be crucial to validate the present findings and to establish reliable imaging endpoints for future DS AD trials.

## Conclusion

In conclusion, age-related neurodegeneration in individuals with DS reflects a dual contribution from ageing and AD-like pathology related to loss of associative memory and DS-specific separable neurodevelopmental mechanisms. Atrophy in the parieto-temporal cortex and left hippocampus likely corresponds to hallmark AD processes, signalling early and transitional stages of disease progression. Conversely, widespread cortical atrophy extending into regions typically spared in early AD, along with pronounced right-hemispheric susceptibility represents distinctive DS-related ageing features. These findings suggest that while AD pathology drives disease-typical regional changes, DS-specific vulnerabilities lead to atypical and diffuse cortical involvement. Integrating these patterns into biomarker strategies may enhance early detection and improve monitoring of neurodegenerative trajectories in this population.

## Supplementary Material

fcag269_Supplementary_Data

## Data Availability

The data supporting the current findings are available from the corresponding author upon reasonable request. The in-house MATLAB script used to extract mean GM volumes in the ROIs of interest is publicly available in an online repository at the following link: https://github.com/ricardomar/Lilia-journal-2026.
