# Supplementary material for: Distinct brain regions are affected by neurodevelopmental or pre-dementia changes in Down syndrome
Source: Brain Commun. 2026 Jul 13;8(4):fcag269. doi: 10.1093/braincomms/fcag269 (PMC13390646; doi:10.1093/braincomms/fcag269)
Supplement: fcag269_Supplementary_Data [file fcag269_supplementary_data.docx]

**SUPPLEMENTARY MATERIAL**

**Participants**

**CONSORT-style flow diagram for participants with Down syndrome**


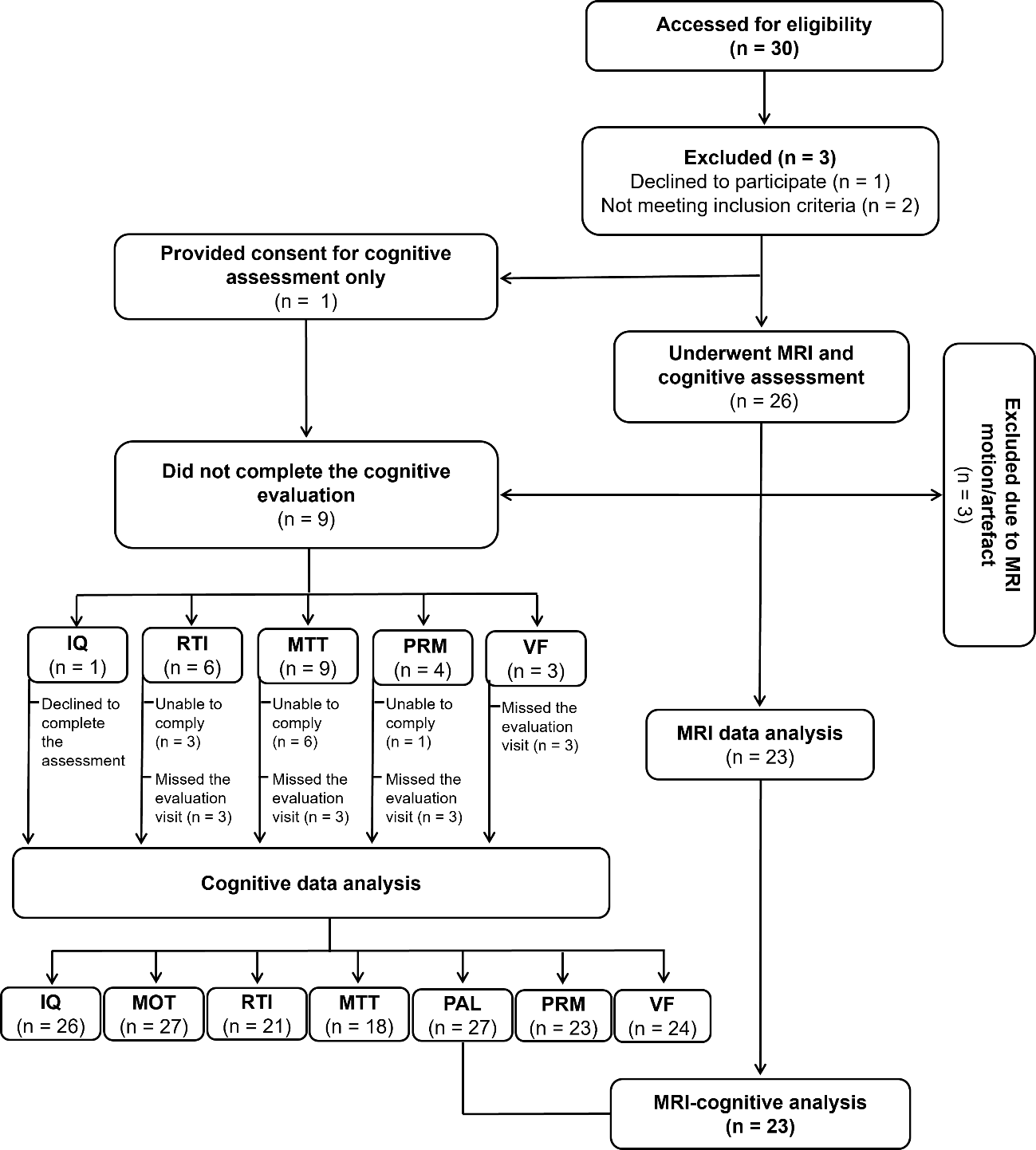


**Supplementary Figure 1** CONSORT‑style flow diagram summarizing recruitment, screening, exclusion criteria, and the final number of participants with Down Syndrome included in each analysis. n, number of participants; MRI, magnetic resonance imaging; IQ, intelligence quotient; MOT, motor screening task; RTI, reaction time; MTT, multitasking test; PAL, paired associates learning; PRM, pattern recognition memory; VF, verbal fluency.

**Neuropsychological assessment (description of the testes used**)

**Supplementary Table 1** List of the neuropsychological tests used to access different cognitive domains

| Cognitive domain | Test | Main ability assessed | Description | Outcomes/ ranges |
| --- | --- | --- | --- | --- |
| Intelligence Quotient (IQ) | WAIS - III | General cognitive abilities | Verbal and Performance subtests. | mean = 100  SD =15 |
| Memory | CANTAB  Paired Associates Learning  (PAL) | Visual episodic memory | Select the correct box (the patterns original location) for a target (pattern), which was previously shown. | First attempt memory score  (0 – 20) |
|  | CANTAB Pattern Recognition Memory (PRM) | Visual pattern recognition memory | From a dual-choice pattern stimulus, choose the correct one previously seen in detriment of a novel pattern. | Percent correct Immediate (0 – 100) |
| Executive function | CANTAB Multitasking Test (MTT) | Executive functions | During this cognitive demanding task, participants have to deal with conflicting information (arrow’s direction or its location on the screen), according with the given instruction. | Total incorrect  (0 – 160) |
|  | Verbal fluency test (VF) | Semantic fluency | Animals and Food categories. | 1 point per each well generated word |
| Sensorimotor | CANTAB Motor Screening Task (MOT) | Sensorimotor screening, and comprehension assurance | Press a cross which appears in different locations on the screen as rapidly and precisely as possible. | Mean latency  (0 – 6000 ms) |
| Attention and Processing speed | CANTAB Reaction Time (RTI) | Motor and mental response speeds | Select and press a response  button on the bottom of the screen and react (releasing it), quickly when one of the five circles turns yellow on the top of the screen, tapping that target stimulus. | Median five-  choice reaction time  (100 -5100 ms) |
| Dementia | DSQIID | Screening for dementia | Screening questionnaire directed to carer’s DS with a set of symptoms of dementia | Screening cut-off >= 20 (possible, but not definite) Dementia |

DSQIID: Dementia Screening Questionnaire for Individuals with Intellectual Disabilities; SD: standard deviation; WAIS-III: Wechsler Adult Intelligence Scale-Third Edition.

## **Results**

##

## **Demographical and Neuropsychological data**

**Supplementary Table 2** Demographic characteristics of the group with DS and TD, along with the results of the between‑group statistical comparisons

|  | | DS group (n = 23) | TD group (n = 24) | P |
| --- | --- | --- | --- | --- |
| Age | **Mean ± SD** | 34.83 ± 12.33 | 32.42 ± 11.42 | 0.502^a^ |
|  | **Median** | 32 | 30.5 |  |
| Female/Male ratio | | 5/18 | 8/16 | 0.517^b^ |

DS, Down syndrome; TD, typically developed; SD, standard deviation; n, number of participants.

^a^Tested with two-sample t-test. ^b^ Tested with Fisher's exact test. Two‑tailed tests were used, and P < 0.05 was considered statistically significant.


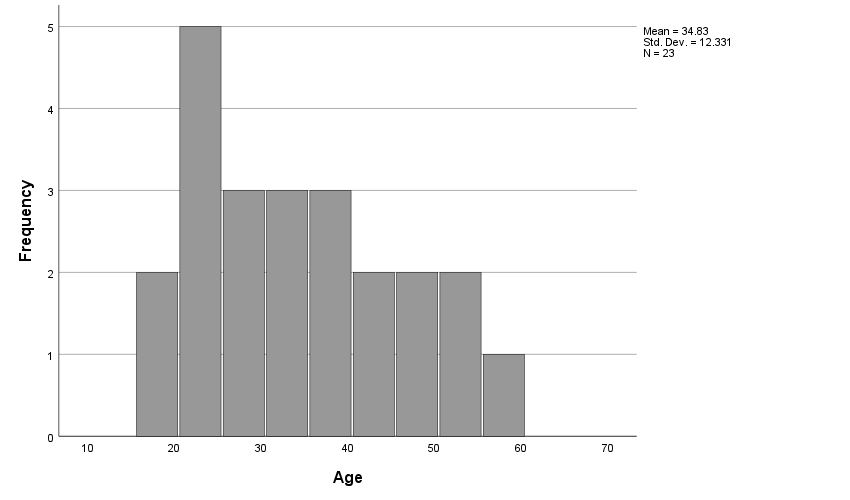


**Supplementary Figure 2** Age distribution of the participants with DS (n = 23) included in the MRI and MRI-cognition analyses. DS, Down syndrome, MRI, magnetic resonance imaging; n, number of participants.

**Supplementary Table 3** Neuropsychological characteristics of participants with DS and its associations with age

| Cognitive test | n | Min – max | Mean  (± SD) | Correlation with age (r, P ^a^) |
| --- | --- | --- | --- | --- |
| IQ (WAIS-III) | 26 | 45 – 66 | 53.15 ± 5.17 | 0.69, p < 0.001 |
| MOT  Mean latency | 27 | 720.30 – 3354.10 | 1382.09 ± 597.30 | 0.65, p < 0.001 |
| RTI  Median five-  choice reaction time | 21 | 377 – 1198 | 556.57 ± 168.51 | 0.40, p = 0.057 |
| MTT  Total incorrect output | 18 | 20 – 70 | 41.61 ± 12.92 | -0.040, p = 0.88 |
| PAL  First attempt memory score | 27 | 0 – 18 | 5.59 ± 4.60 | -0.46, p = 0.016 |
| PRM  Percent correct immediate | 23 | 16.67 – 100 | 60.04 ± 22.52 | -0.27, p = 0.21 |
| Verbal Fluency test | 24 | 2 – 25 | 15.21 ± 6.58 | -0.026, p = 0.90 |

^a^ Pearson correlation, a threshold of P < 0.05 (uncorrected for multiple comparisons) was used to determine statistical significance; n, number of participants; SD, standard deviation; IQ, intelligence quotient; MOT, motor screening task; MTT, multitasking test; PAL, paired associates learning; PRM, pattern recognition memory; RTI, reaction time. Missing data: IQ = 1 (declined to complete the assessment); RTI = 6 (3 unable to comply with the task; 3 missed the evaluation visit); MTT = 9 (6 unable to comply with the task; 3 missed the evaluation visit); PRM = 4 (1 unable to comply with the task; 3 missed the evaluation visit); verbal fluency = 3 (missed the evaluation visit).

**Between-groups comparison of age-related structural patterns in overall DS-reduced regions**

**Supplementary Table 4** Age-related group differences in clusters showing reduced GM volume in individuals with DS

| Region | Side | Group | Slope | Difference from zero  P | Slope differences between groups | |
| --- | --- | --- | --- | --- | --- | --- |
|  |  |  |  |  | **F (1, 43)** | **P ^a^** |
| Cerebellum | L/R | DS | -9.36e-0.007 | 0.0022 | 1.029 | 0.316 |
|  |  | TD | -3.81e-0.007 | 0.44 |  |  |
| Orbitofrontal  (BA 11/25) | L/R | DS | -1.01e-0.006 | 0.0046 | 0.0035 | 0.953 |
|  |  | TD | -9.85e-0.007 | 0.013 |  |  |
| Anterior cingulate  (BA 10/23) | R | DS | -1.44e-0.006 | 0.0023 | 0.87 | 0.357 |
|  |  | TD | -2.01e-0.006 | < 0.001 |  |  |
| Temporal  (BA 22/13) | L | DS | -1.59e-0.006 | 0.016 | 0.0052 | 0.942 |
|  |  | TD | -1.64e-0.006 | < 0.001 |  |  |
|  | R | DS | -1.71e-0.006 | 0.004 | 0.0028 | 0.958 |
|  |  | TD | -1.74e-0.006 | < 0.001 |  |  |
| Hippocampus | L | DS | -1.45e-0.006 | 0.011 | 4.41 | 0.042 |
|  |  | TD | 6.31e-0.008 | 0.90 |  |  |
|  | R | DS | -1.20e-0.006 | 0.016 | 1.57 | 0.217 |
|  |  | TD | -3.39e-0.007 | 0.52 |  |  |

GM, grey matter, BA, Brodmann area; DS, Down syndrome; TD, typically developed; L, left; R, right; n, number of participants.

Results of the between-groups comparison (DS group, n = 23; TD group, n = 24) of the GM-versus-age regression slopes in DS-reduced regions obtained in GraphPad prism through an equivalent ANCOVA procedure. A threshold of ^a^ P < 0.05 (uncorrected for multiple comparisons) was used to determine statistical significance. Plots of the linear regressions are shown in Figure 2.

**Between-groups comparison of age-related structural patterns in hippocampal subfields delineated using the CoBrA anatomical atlas**











**Supplementary Figure 3** Relationship between hippocampal subfields GM volume and age in the group with DS (n = 23) and TD (n = 24) group. The plot shows linear regressions of mean GM volume (normalized to TIV) as a function of age across bilateral hippocampal subfields defined by the CoBrA anatomical atlas: **(A)** CA1, **(B)** CA2/CA3, **(C)** CA4/dentate gyrus, **(D)** subiculum, and **(E)** stratum radiatum. Each point represents an individual participant’s mean GM volume within the corresponding subfield. While hippocampal subfield volumes appear broadly comparable between DS and TD participants at younger ages, the DS group exhibits a steeper age-related decline, suggesting more pronounced structural deterioration in later adulthood. Statistics from the linear regressions are detailed in Supplementary Table 5. Values on the y-axes are normalized to TIV, which accounts for their magnitude and renders them unitless. DS, Down syndrome; TD, typically developed; GM, grey matter; TIV, total intracranial volume; L, left; R, right; n, number of participants; Hipp., hippocampus. CA1, cornu ammonis 1; CA2/CA3; cornu ammonis 2 and 3; CA4, cornu ammonis 4; SR/SL/SM; stratum radiatum/stratum lacunosum/stratum moleculare.

**Supplementary Table 5** Age‑related group differences in GM volume across hippocampal subfields

| Region | Side | Group | Slope | Difference from zero  P | Slope differences between groups | |
| --- | --- | --- | --- | --- | --- | --- |
|  |  |  |  |  | **F (1,43)** | **P ^a^** |
| CA1 | L | DS | -1.93e-006 | < 0.001 | 10.21 | 0.0027 |
|  |  | TD | 1.62e-0.007 | 0.72 |  |  |
|  | R | DS | -1.99e-0.006 | < 0.001 | 9.90 | 0.0030 |
|  |  | TD | 4.47e-0.008 | 0.92 |  |  |
| CA2/CA3 | L | DS | -1.70e-0.006 | 0.012 | 4.88 | 0.033 |
|  |  | TD | 1.14e-0.007 | 0.83 |  |  |
|  | R | DS | -1.912e-0.006 | 0.0075 | 4.17 | 0.047 |
|  |  | TD | -2.867e-0.007 | 0.54 |  |  |
| CA4/dentate gyrus | L | DS | -1.74e-0.006 | 0.0034 | 6.78 | 0.013 |
|  |  | TD | 1.587e-0.007 | 0.75 |  |  |
|  | R | DS | -1.92e-0.006 | 0.0027 | 6.55 | 0.014 |
|  |  | TD | 7.25e-0.009 | 0.99 |  |  |
| Subiculum | L | DS | -1.32e-0.006 | 0.0057 | 8.51 | 0.0056 |
|  |  | TD | 4.64e-0.007 | 0.30 |  |  |
|  | R | DS | -1.43e-0.006 | < 0.001 | 10.33 | 0.0025 |
|  |  | TD | 2.69e-0.007 | 0.50 |  |  |
| SR/SL/SM | L | DS | -1.66e-0.006 | 0.0031 | 7.33 | 0.0097 |
|  |  | TD | 2.21e-0.007 | 0.65 |  |  |
|  | R | DS | -1.67e-0.006 | 0.0021 | 6.88 | 0.012 |
|  |  | TD | 6.81e-0.008 | 0.88 |  |  |

GM, grey matter; DS, Down syndrome; TD, typically developed; CA1, cornu ammonis 1; CA2/CA3; cornu ammonis 2 and 3; CA4, cornu ammonis 4; SR/SL/SM; stratum radiatum/stratum lacunosum/stratum moleculare; L, left; R, right; n, number of participants.

Results of the between-groups comparison (DS group, n = 23; TD group, n = 24) of the GM-versus-age regression slopes in hippocampal subfields obtained in GraphPad prism through an equivalent ANCOVA procedure. A threshold of ^a^ P < 0.05 (uncorrected for multiple comparisons) was used to determine statistical significance. Plots of the linear regressions are shown in Supplementary Figure 3.

**Sensitivity analysis in the hippocampus subfields using a smaller smoothing kernel (6 mm FWHM)**

**

**

**

**

**

**

**Supplementary Figure 4** Relationship between hippocampal subfields GM volume and age in the group with DS (n = 23) and TD (n = 24) group, after reprocessing the GM data with a 6 mm FWHM gaussian filter. The plot shows linear regressions of mean GM volume (normalized to TIV) as a function of age across bilateral hippocampal subfields defined by the CoBrA anatomical atlas: **(A)** CA1, **(B)** CA2/CA3, **(C)** CA4/dentate gyrus, **(D)** subiculum, and **(E)** stratum radiatum. Each point represents an individual participant’s mean GM volume within the corresponding subfield. While hippocampal subfield volumes appear broadly comparable between DS and TD participants at younger ages, the DS group exhibits a steeper age-related decline, suggesting more pronounced structural deterioration in later adulthood. Statistics from the linear regressions are detailed in Supplementary Table 6. Values on the y-axes are normalized to TIV, which accounts for their magnitude and renders them unitless. DS, Down syndrome; TD, typically developed; GM, grey matter; FWHM, Full Width at Half Maximum; TIV, total intracranial volume; L, left; R, right; n, number of participants; Hipp., hippocampus. CA1, cornu ammonis 1; CA2/CA3; cornu ammonis 2 and 3; CA4, cornu ammonis 4; SR/SL/SM; stratum radiatum/stratum lacunosum/stratum moleculare.

**Supplementary Table 6** Results of the sensitivity analysis across hippocampal subfields using GM data smoothed with a 6 mm FWHM filter

| Region | Side | Group | Slope | Difference from zero  P | Slope differences between groups | |
| --- | --- | --- | --- | --- | --- | --- |
|  |  |  |  |  | **F (1,43)** | **P ^a^** |
| CA1  (6 mm) | L | DS | -1.93e-0.006 | 0.0011 | 10.20 | 0.0026 |
|  |  | TD | 2.72e-0.007 | 0.56 |  |  |
|  | R | DS | -2.00e-0.006 | 0.0010 | 9.37 | 0.0038 |
|  |  | TD | 1.41e-0.007 | 0.76 |  |  |
| CA2/CA3  (6 mm) | L | DS | -1.68e-0.006 | 0.038 | 2.85 | 0.099 |
|  |  | TD | 3.27e-0.008 | 0.96 |  |  |
|  | R | DS | -2.15e-0.006 | 0.013 | 3.38 | 0.073 |
|  |  | TD | -3.71e-0.007 | 0.51 |  |  |
| CA4/dentate gyrus  (6 mm) | L | DS | -1.70e-0.006 | 0.0092 | 5.13 | 0.029 |
|  |  | TD | 1.49e-0.007 | 0.79 |  |  |
|  | R | DS | -2.01e-0.006 | 0.0045 | 5.97 | 0.019 |
|  |  | TD | 6.33e-0.008 | 0.911 |  |  |
| Subiculum  (6 mm) | L | DS | -1.49e-0.006 | 0.0044 | 10.41 | 0.0023 |
|  |  | TD | 6.72e-0.007 | 0.18 |  |  |
|  | R | DS | -1.51e-0.006 | < 0.001 | 12.49 | < 0.001 |
|  |  | TD | 3.99e-0.007 | 0.34 |  |  |
| SR/SL/SM  (6 mm) | L | DS | -1.58e-0.006 | 0.0077 | 6.18 | 0.017 |
|  |  | TD | 2.91e-0.007 | 0.59 |  |  |
|  | R | DS | -1.70e-0.006 | 0.0037 | 6.39 | 0.015 |
|  |  | TD | 1.56e-0.007 | 0.77 |  |  |

GM, grey matter; FWHM, Full Width at Half Maximum; DS, Down syndrome; TD, typically developed; L, left; R, right; n, number of participants; CA1, cornu ammonis 1; CA2/CA3; cornu ammonis 2 and 3; CA4, cornu ammonis 4; SR/SL/SM; stratum radiatum/stratum lacunosum/stratum moleculare.

Results of the between-groups comparison (DS group, n = 23; TD group, n = 24) of the GM-versus-age regression slopes in hippocampal subfields (6 mm‑smoothed GM data) obtained in GraphPad prism through an equivalent ANCOVA procedure. A threshold of ^a^ P < 0.05 (uncorrected for multiple comparisons) was used to determine statistical significance. Plots of the linear regressions are shown in Supplementary Figure 4.

## **Age-related effects on whole brain GM volume in group with DS and TD group**

**Supplementary Table 7** Cerebral clusters demonstrating significant associations between GM volume and age in participants with DS and TD

|  |  | Cluster-Level | | Peak voxel-level | | | | | |
| --- | --- | --- | --- | --- | --- | --- | --- | --- | --- |
| Brain Region | **Side** | **Cluster**  **size** | **qFDR ^a^** | **MNI coordinates** | | | **T** | **Equi-Z** | **P ^b^** |
|  |  |  |  | **x** | **y** | **z** |  |  |  |
| DS group (n = 23) | | | | | | | | | |
| 1 - Supramarginal gyrus/ angular gyrus (BA 40/39) | R | 2693 | < 0.001 | 60 | -23 | 35 | 7.97 | 5.35 | < 0.001 |
| 2 - Fusiform gyrus/ middle and inferior temporal gyrus (BA 37/21/20) / cerebellum | R | 2659 | < 0.001 | 44 | -51 | -23 | 7.77 | 5.28 | < 0.001 |
| 3 - Superior and middle temporal gyrus / angular gyrus (BA 22/21/39) | R | 1873 | < 0.001 | 63 | -41 | 9 | 7.47 | 5.16 | < 0.001 |
| 4 - PCC/ precuneus  (BA 31/ 7) | L/R | 6154 | < 0.001 | -3 | -51 | 42 | 7.22 | 5.06 | < 0.001 |
| 5 - Lingual and fusiform gyrus (BA 37) / cerebellum / hippocampus | L | 1788 | 0.003 | -21 | -50 | -8 | 7.13 | 5.03 | < 0.001 |
| 6 - Angular gyrus/ supramarginal gyrus  (BA 39/40) | L | 603 | 0.002 | -45 | -63 | 48 | 6.66 | 4.83 | < 0.001 |
| 7 - Superior and middle temporal gyrus (BA 22/21) | L | 642 | < 0.001 | -60 | 2 | -14 | 6.55 | 4.78 | < 0.001 |
| 8 - Middle and superior temporal gyrus/ fusiform gyrus/ parahippocampal gyrus (BA 21/22/37/36) / hippocampus | R | 2232 | < 0.001 | 44 | -5 | -23 | 6.55 | 4.78 | < 0.001 |
| 9 - Inferior temporal gyrus  (BA 20) / cerebellum | L | 386 | 0.018 | -45 | -45 | -27 | 6.41 | 4.72 | < 0.001 |
| 10 - Orbitofrontal (BA11) | L | 351 | 0.024 | -9 | 26 | -23 | 6.36 | 4.70 | < 0.001 |
| 11 - Insula (BA13) | L | 487 | 0.007 | -42 | -14 | 8 | 5.81 | 4.44 | < 0.001 |
| 12 - Anterior PFC (BA10) | L | 800 | 0.001 | -21 | 54 | -9 | 5.80 | 4.43 | < 0.001 |
| *13 - DLPFC* (BA9) | R | 328 | 0.028 | 20 | 48 | 27 | 5.63 | 4.35 | < 0.001 |
| TD group (n = 24) | | | | | | | | | |
| Superior temporal gyrus/ insula (BA 22/13) | R | 980 | < 0.001 | 50 | -2 | -5 | 8.47 | 5.59 | < 0.001 |
| Superior temporal gyrus/ insula (BA 22/13) | L | 477 | 0.016 | -47 | -8 | -5 | 7.66 | 5.29 | < 0.001 |
| Cingulate cortex (BA 24/32) | R | 1245 | < 0.001 | 2 | 8 | 24 | 6.46 | 4.79 | < 0.001 |

GM, grey matter; DS, Down syndrome; TD, typically developed; BA, Brodmann area; MNI, Montreal Neurological Institute space; L, left; R, right; n, number of participants; PCC, posterior cortical cortex; PFC, prefrontal cortex; DLPFC, dorsolateral prefrontal cortex;

Results of the regression voxel-wise GM-age regression analysis in group with DS and TD group obtained with an initial voxel-wise P < 0.0001 uncorrected, cluster ≥ 150 voxels, followed by cluster‑level correction for multiple comparisons qFDR ≤ 0.05. ^a^ FDR‑corrected cluster‑level *q-*value. ^b^ Uncorrected peak-level *P*‑value.

## **Effects of cognitive measures on GM volume**

**Supplementary Table 8** Brain regions showing positive associations between GM volume and episodic memory (PAL first attempt memory score) in the DS group (n = 23)

| Brain Region | Side | Cluster-Level | Peak-level | | | | | |
| --- | --- | --- | --- | --- | --- | --- | --- | --- |
|  |  | **Cluster size** | **MNI coordinates** | | | **T** | **Equi-Z** | **P ^a^** |
|  |  |  | **x** | **y** | **z** |  |  |  |
| Angular gyrus/ supramarginal/ middle temporal gyrus  (BA 39/40/21) | L | 4066 | -51 | -60 | 18 | 5.57 | 4.31 | < 0.001 |
| Middle temporal gyrus (BA 21) | R | 1987 | 68 | -41 | -12 | 4.33 | 3.62 | < 0.001 |
| Precuneus/ precentral gyrus/ PCC (BA 7/4/31) | R/L | 2237 | 8 | -32 | 56 | 4.27 | 3.58 | < 0.001 |
| Ventrolateral PFC/ orbitofrontal cortex  (BA 47/11) | L | 446 | -17 | 32 | -20 | 3.38 | 2.99 | 0.0014 |
| Angular Gyrus (BA 39) | R | 411 | 45 | -68 | 44 | 3.25 | 2.89 | 0.0019 |

GM, grey matter; PAL, Paired Associates Learning; DS, Down syndrome; BA, Brodmann area; PCC, posterior cortical cortex; PFC, prefrontal cortex; L, left; R, right; MNI, Montreal Neurological Institute space; n, number of participants.

Results of the regression voxel-wise GM-PAL regression exploratory analysis in DS obtained with a voxel- wise P < 0.01 (uncorrected), cluster ≥ 320 voxels. ^a^ Uncorrected peak-level *P*‑value.
